# AI-Powered Chatbots in Medical Education: Potential Applications and Implications

**DOI:** 10.7759/cureus.43271

**Published:** 2023-08-10

**Authors:** Nima Ghorashi, Ahmed Ismail, Pritha Ghosh, Anton Sidawy, Ramin Javan

**Affiliations:** 1 Department of Radiology, George Washington University School of Medicine and Health Sciences, Washington, USA; 2 Department of Neurology, George Washington University School of Medicine and Health Sciences, Washington, USA; 3 Department of Surgery, George Washington University School of Medicine and Health Sciences, Washington, USA

**Keywords:** medical education, chatbots, large language models, artificial intelligence, gpt-4, chatgpt

## Abstract

Artificial intelligence (AI) is anticipated to have a considerable impact on the routine practice of medicine, spanning from medical education to clinical practice across specialties and, ultimately, patient care. With the imminent widespread adoption of AI in medical practice, it is imperative that medical schools adapt to the use of these advanced technologies in their curriculum to produce future healthcare professionals who can seamlessly integrate these tools into practice. Chatbots, AI systems programmed to process and generate human language, are currently being evaluated for various tasks in medical education. This paper explores the potential applications and implications of chatbots in medical education, specifically in learning and research. With their capability to summarize, simplify complex concepts, automate the creation of memory aids, and serve as an interactive tutor and point-of-care medical reference, chatbots have the potential to enhance students' comprehension, retention, and application of medical knowledge in real-time. While the integration of AI-powered chatbots in medical education presents numerous advantages, it is crucial for students to use these tools as assistive tools rather than relying on them entirely. Chatbots should be programmed to reference evidence-based medical resources and produce precise and trustworthy content that adheres to medical science standards, scientific writing guidelines, and ethical considerations.

## Editorial

Introduction

**Figure 1:**
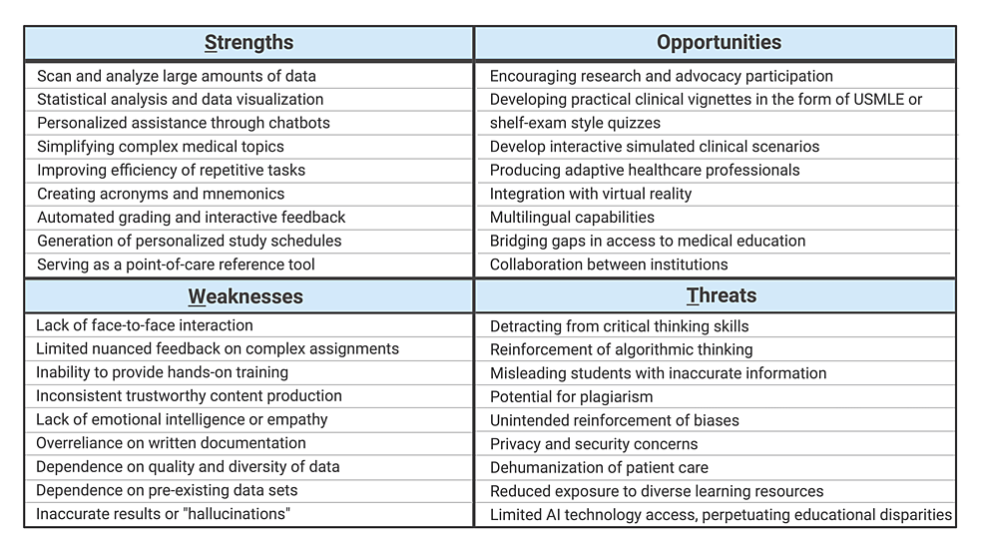
SWOT Analysis Applications and implications for the use of AI-powered chatbots in medical education.

The healthcare industry is on the verge of experiencing a transformation in which artificial intelligence (AI) is anticipated to impact the routine practice of medicine, spanning from medical education to clinical practice across specialties and, ultimately, patient care [1]. It is imperative that medical schools adapt to the use of these advanced technologies in their curriculum to produce future healthcare professionals who can seamlessly integrate these tools into practice. Medical students have expressed interest in revising the medical curriculum to adapt to the changing healthcare environment influenced by AI [2]. Institutions have initiated efforts to explore the integration of emerging AI-related topics into their educational programs [3]. Furthermore, medical school administrations have already begun hosting national workshops focused on AI in health professions education to acquire a comprehensive understanding of how these advanced technologies function as well as their implications and applications [4]. Chatbots are AI systems programmed to understand, process, and generate human language, pre-trained on specific input data to respond to a wide range of queries more effectively, and retrieve information from the internet quickly and accurately using their advanced natural language processing (NLP) model. With the increasing interest and exploration for the utilization of AI-powered chatbots in medical education, we present possible applications and implications associated with their adoption, and propose a brief preliminary SWOT (strengths, weaknesses, opportunities, and threats) analysis (Figure 1).

Medical education

Preclinical Interactive Tutor and Learning Tool

Chatbots can serve as interactive tutors and learning tools for students. In their current state, chatbots can provide a basic level of tailored support, such as acting as an interactive search engine to answer questions that arise from the learner, offering real-time feedback, and guiding students through challenging concepts. This enables students to receive personalized assistance outside of the classroom. Additionally, current chatbots can automate repetitive, time-consuming tasks such as transforming lecture material into question- and answer-based flashcards instantly, further facilitating active recall and retention of information. They can also assist with developing acronyms and mnemonics for diagnoses with key clinical findings or criteria and summarize these topics in simplified language.

In the future, customizable chatbots can be trained to present clinical scenarios that challenge students to work through simulated patient presentations, clinical management, decision-making skills, and responding appropriately to complications. These scenarios may be designed to emulate actual medical situations, providing students with a safe and controlled environment to hone their clinical skills. Personalized feedback can provide targeted resources to address areas where students may need improvement.

Chatbots can be a useful tool for educators as well. Although chatbots have the ability to be pre-trained on input data, such as evidence-based resources and clinical teaching files, current chatbots are limited in their ability to consistently produce reliable and effective standardized board-style questions or simulated clinical assessments. However, future customizable chatbots should focus on their ability to create clinical vignettes in the form of USMLE or shelf-exam style quizzes. By using chatbots to create these quizzes, students can receive interactive and instant feedback on their performance, further probe incorrect answer choices, and gain a better understanding of their strengths and weaknesses in various subject areas.

Clinical Ward Assistant 

Currently, publicly accessible chatbots may not be sufficiently dependable for use by trainees in clinical settings due to their reliance on a wide array of open internet sources. However, current chatbots have the capability to be programmed to extract information from evidence-based resources and present it in a natural conversational language format. Thus, in the future, chatbots can serve as a useful point-of-care reference that provides clinical information in an understandable, efficient, interactive, and scenario-specific format. This can assist students in comprehending complex medical topics and developing clinical decision-making skills. Further, this technology can serve as quick references for medication use indications, dosages, drug-drug interactions, and regimens for different diagnoses. By providing summaries, simplifying complex concepts, and offering memory aids, chatbots have the potential to enhance students' comprehension, retention, and application of medical knowledge in real-time. 

Although publicly available chatbots can currently be programmed to generate medical notes or assessments, it is important to emphasize that the responsibility for creating history and physical examination (H&P) medical notes or narrative assessments and plans lies with the individual student. As this technology advances and becomes integrated into electronic medical records, future chatbots can be a useful tool to assist students in the generation of these documents for efficiency and consistency, but they should not be used to replace critical thinking and decision-making skills necessary to accurately document a patient's medical history, physical examination findings, and to develop an appropriate assessment and plan. 

Chatbots have the potential to enhance medical trainee engagement in patient education, aiding tasks such as generating health-related articles for bedside, clinic, and take-home use. These chatbots can be pre-trained on complex medical literature to produce evidence-based, customizable, simplified materials that are adaptable to different languages and medical terminology levels. Current publicly available chatbots have the capability to generate these educational materials with relevant image creation for unique patient-specific scenarios and translate them into countless different languages in real time. However, future chatbots for this purpose should reference evidence-based resources and continue to improve translation capabilities for proficiency and accuracy. This technology facilitates rapid, effective patient education, streamlining information acquisition and content creation for bedside, outpatient, and post-visit references. Further, it is important for trainees to realize that similar technology can be developed for patient use in the outpatient setting to answer health-related questions, as this can directly impact the landscape of patient care.

Important Considerations

Chatbots cannot fulfill the human experience and have potential biases intertwined within the practice of medicine. Medical education involves hands-on experience, direct patient care, and the understanding of non-verbal cues, all of which cannot be fully simulated by chatbots. This technology is only as effective as their programming and the data they are trained on. If the chatbot's input data is incomplete, insufficient, or biased, it may not provide accurate information to learners and can misguide patient care. Lastly, and this cannot be overstated, not all students may have access to chatbot technology. Limited access can further perpetuate educational disparities and outcomes. Regulatory bodies need to be formed to monitor the development and implementation of this technology.

Continuing education and research

Keeping Up With Medical Literature 

Keeping pace with the evolving nature of health sciences education can be challenging for health sciences students; however, it is crucial for trainees at all levels to stay up to date with the latest research and advancements. Current AI text classifiers can play a crucial role in classifying and categorizing research articles, facilitating researchers to locate relevant research articles based on their research topics, and organizing and classifying vast amounts of scientific literature into simplified text. Future customized chatbots for medical education can integrate AI text classifiers for trainees and educators to stay up-to-date with the latest developments in different fields by identifying relevant articles on specific topics and creating summaries, which can be valuable for quick reference and spurring particular research interests. 

Scientific Process

Undertaking research and initiating a research project can be daunting and overwhelming for health sciences students, but chatbots can help facilitate this process and encourage greater participation in research among students. With the current ability of publicly available chatbots to scan existing research through the use of filtering parameters such as publication date, author, and research topic, they can aid in several essential tasks, such as assisting with early stages of literature reviews by collating relevant peer-reviewed articles, journals, and databases based on specific research criteria and identifying trends and future directions in the field, which can help formulate potential research topics, initial hypotheses, and potential subtopic discussions. However, in its current state, publicly available chatbot technology lacks the ability to conduct comprehensive literature searches or engage in critical analysis and discussion of articles [5]. These functions can help medical students overcome the intimidating task of starting research and allow them to more efficiently develop research questions and directions that align with current research and advancements.

Statistical Analyses

The limited statistical analysis training among health sciences students as a component of the standard medical curriculum is a noteworthy barrier to research engagement. Currently available chatbots can analyze appropriately input data, recommend suitable statistical methods that align with the research question and the nature of the data, and create graphical representations of data and results for poster presentations and publications. This can enhance the accuracy and efficiency of data analysis, enhance the communication of research findings, and ultimately improve the validity of research outcomes for students.

Important Considerations

The integration of AI-powered chatbots in research presents numerous advantages, but it is crucial for students to use these tools as assistive tools rather than relying on them entirely. Future customized chatbots for medical education should be programmed to reference evidence-based medical resources and produce precise and trustworthy content that adheres to medical science standards, scientific writing guidelines, and ethical considerations, keeping in mind that current publicly available chatbots have the potential to falsify and create references, a concept known as “hallucination”. Chatbots should clearly cite the sources used in their responses to ensure that students are not misled by inaccurate or incomplete information and that they are utilizing reliable, peer-reviewed sources. AI algorithms that detect chatbot-generated text may help institutions mitigate plagiarism, encourage originality, and maintain academic thinking, although a threshold of acceptable use must be clearly established.

Health policy and advocacy

AI-powered chatbots can serve an important role in increasing the involvement of medical trainees and educators in health policy, advocacy, and public health by supporting various tasks related but not limited to preventive health campaigns, evidence-based health-related articles, communication with politicians, and lobbying efforts. For example, chatbots can be pre-trained on complex medical references and generate simplified text to educate patients and the public about preventive imaging campaigns, emphasizing the significance of mammography or colonoscopy. They can provide information on the screening process, answer frequently asked questions, and facilitate appointment scheduling. These chatbots can also gather feedback on the effectiveness of campaigns, identify areas for improvement, and modify their programming accordingly.

Another promising application is their ability to facilitate communication with lawmakers regarding important health policy issues. They can be programmed to analyze vast amounts of peer-reviewed medical literature and provide summaries to the public and legislative officials in the form of regularly distributed, evidence-based, concise, and understandable articles and blogs on specific legislation, presenting different positions with data and research. Furthermore, they can assist in writing letters and emails to lawmakers on behalf of medical trainees. By automating certain tasks, such as identifying key stakeholders, scheduling meetings, and sending follow-up communications, chatbots can help streamline lobbying efforts. They can also analyze data related to lobbying efforts and provide valuable insights into areas that require more attention or resources. Additionally, chatbots can be used to develop persuasive arguments and messaging to support lobbying efforts. With these capabilities, AI-powered chatbots can potentially increase medical trainee involvement and enhance the way health policy, advocacy, and public health efforts are conducted by enabling faster and more efficient communication, information gathering, and analysis.

Important Considerations

While AI-powered chatbots can analyze vast amounts of medical literature, they may not always accurately interpret, contextualize, and apply the findings. Secondly, personal relationships and face-to-face interactions with lawmakers and stakeholders can often be more persuasive than written communication. Simply put, chatbots lack the key human element necessary for successful lobbying efforts. Overall, chatbots can certainly play a helpful role in supporting health policy, advocacy, and public health efforts, but they should not be seen as a substitute for the expertise and guidance of medical professionals and the importance of face-to-face communication.

To move closer to using AI-powered chatbots in medical education, several steps need to be taken. First, frameworks and tools must be established to integrate chatbots into existing medical education programs. This may involve developing chatbot platforms specifically for medical education and creating content that aligns with the standardized curriculum and learning objectives. Second, the potential benefits and limitations of using chatbots in medical education must be further examined. While chatbots have the potential to improve student engagement, enhance learning outcomes, and provide personalized support, there may also be concerns about data privacy, the accuracy of the information provided, overreliance on these tools, and the impact on interpersonal communication skills. To address these concerns, efforts should be made to ensure that chatbots are designed and implemented in a way that is both ethical and effective. This may involve establishing guidelines and best practices for chatbot design and usage, training medical educators and students on how to use chatbots effectively, and conducting rigorous evaluations of chatbot performance and impact. Furthermore, it is essential to involve stakeholders in the development and implementation of chatbots in medical education, including students, educators, providers, developers, and policymakers. This will help ensure that the needs and concerns of all parties are considered and that chatbots are developed and implemented in a way that is both beneficial and acceptable to all stakeholders. With the appropriate frameworks, tools, and safeguards in place, AI-powered chatbots have the potential to transform medical education and research.
